# Mediator Selection in Linear and Nonlinear Mediation Analysis via Adaptive Measurement Errors Infusion

**DOI:** 10.21203/rs.3.rs-11297719/v1

**Published:** 2026-10-27

**Authors:** Chen Liang, Wen Zhou, Mengling Liu

**Affiliations:** 1 Division of Biostatistics, Department of Population Health, NYU Grossman School of Medicine, New York, 10016, NY, USA.; 2 Department of Biostatistics, School of Global Public Health, New York University, New York, 10003, NY, USA.

**Keywords:** Kernel regression, measurement error modeling, multiple mediators, nonlinear mediation

## Abstract

**Background:**

Mediation analysis investigates intermediate mechanisms through which an exposure influences an outcome. When there are multiple candidate mediators, mediator selection should precede the estimation of mediation effects but remains challenging when pathway signals are weak or imbalanced, when mediator-outcome relationships are nonlinear, and when there are mediator-exposure and/or mediator-mediator interactions. Most existing methods rely on coefficient-based regularization under linear outcome models. We propose a multiple-mediator selection framework based on the measurement error modeling (MEM) principle, which quantifies a mediator’s importance by how much the fit of the exposure–mediator and mediator–outcome pathways degrades when the mediator is deliberately perturbed by a controlled infusion of artificial measurement error.

**Results:**

For linear mediation systems, the proposed adaptive MEM procedure distributes a constrained measurement-error precision budget across mediators under a joint Gaussian working criterion, then selects exposure–mediator and mediator–outcome pathways separately, and lastly retains a mediator only if it is selected in both pathways. For nonlinear mediator-outcome relationships, an adaptive kernel MEM extension retains linear exposure–mediator models and replaces the error precisions for the mediators in the outcome model with coordinate-specific inverse bandwidths. In simulations, linear MEM was competitive with established methods designed for linear mediation systems, with a modest advantage when mediator residuals were correlated. Kernel MEM substantially improved the selection of mediators entering the outcome model through quadratic or interaction terms relative to the linear methods and outperformed a generic kernel-ridge importance benchmark in most simulation settings. Applied to a DNA methylation study of childhood trauma and adult cortisol stress reactivity, the linear and kernel MEM methods selected eight and three loci, respectively, several of which had been reported in previous analyses of the same data.

**Conclusions:**

The proposed MEM framework provides a practical approach to multiple mediator selection under linear and nonlinear mediator-outcome relationships, yielding candidate mediators supported by evidence from both pathways.

## 1 Introduction

Understanding mediation pathways is essential for studying disease mechanisms and informing prevention and treatment strategies. Mediation analysis provides a principled framework for investigating the intermediate processes through which an exposure affects an outcome, with broad applications in psychology, epidemiology, and genomics (MacKinnon et al. 2007; Richiardi et al. 2013; Zeng et al. 2021). The classical framework of Baron and Kenny (1986) operationalizes mediation analysis through a system of linear regressions, in which the indirect effect is defined as the product of two regression coefficients. More recently, the counterfactual framework has placed mediation analysis on a rigorous causal footing (VanderWeele and Vansteelandt 2009; Valeri and VanderWeele 2013), giving formal definitions of direct and indirect effects, enabling effect decomposition, and making explicit the assumptions under which those effects are identifiable.

Driven by high-throughput omics technologies, mediation analysis with multiple candidate mediators has become increasingly common, and considering multiple pathways simultaneously is necessary both to characterize complex biological mechanisms and to reduce bias from omitted mediators. Therefore, selecting active mediators becomes a key step before estimating mediators’ effects in high-dimensional mediation analysis. The corresponding literature of mediator selection has grown substantially, particularly for linear mediation models (Zeng et al. 2021; Clark-Boucher et al. 2023; Chang et al. 2024), and existing methods differ mainly in how they integrate the information from the exposure–mediator path with that from mediator–outcome path.

One group selects mediators from the exposure–mediator and mediator–outcome models separately, applies penalization techniques to the outcome model, and then combines the evidence afterward. **HI**gh-dimensional **M**ediation **A**nalysis (HIMA) applies the minimax concave penalty (Zhang et al. 2016), **H**igh-**D**imensional **M**ediation **A**nalysis (HDMA) uses the de-sparsified LASSO penalty (Gao et al. 2019), and the **Med**iation Analysis applies **Fix**ed Effect Model (MedFix) uses an adaptive LASSO penalty (Zhang 2021). These procedures are computationally attractive, but relying on the outcome model penalization alone can incur high false negative rates when the mediation signal is weak or unevenly split between the two pathways (Jones et al. 2024). An alternative line of work integrates information from both paths directly. Pathway LASSO minimizes a weighted sum of residual variances from the mediator and outcome models while penalizing both individual coefficients and product-form mediation effects (Zhao and Luo 2022), and the **Reg**ularized **S**tructural **E**quation **M**odel (RegSEM) maximizes a joint likelihood for the mediation system under combined $L_{1}$ and $L_{2}$ regularization (Schaid and Sinnwell 2020). Bayesian formulations, including the **B**ayesian **S**parse **L**inear **M**ixed **M**odel (BSLMM) (Song et al. 2020) and the Gaussian-mixture model (Song et al. 2021), have also been proposed to support mediator selection through hierarchical modeling of pathway coefficients.

Despite these developments, existing selection methods remain largely coefficient-based and rely on strict linear assumptions, limiting their ability to accommodate nonlinear relationships and complex interactions. This is a major limitation for applications where mediator-outcome relationships in molecular data can be driven by threshold effects, quadratic dose-response patterns, or interactions among correlated markers, and a mediator whose relevance depends on such structure will be systematically missed by linear models with coefficient-based penalization regardless of its true biological importance. A selection procedure that remains informative under such flexible mediator-outcome structures is therefore warranted, both to generate biological hypotheses and identify potentially active mechanisms, and to improve downstream estimation by excluding noninformative variables.

In this paper, we introduce a multiple mediator selection framework based on the measurement error modeling (MEM) principle (Stefanski et al. 2014). The MEM-based framework was first developed for variable selection in classification and extended to regression (White et al. 2017; Feng et al. 2018; Lai et al. 2022; Lu et al. 2024). It allocates a fixed budget of measurement-error precision across predictors while minimizing the degradation in model fit. Consequently, informative predictors tend to receive little infused error, whereas uninformative variables absorb large amounts, yielding a mechanism for variable selection in this single-model setting. Extending this property to mediator selection is nontrivial, however, since a mediator’s relevance is jointly determined by two pathways rather than one. Motivated by the MEM idea, we recast mediator selection as a problem of error tolerance across both pathways simultaneously, selecting mediators according to how the fit of the joint mediation system deteriorates when mediators are perturbed by error infusions. For selection in linear mediation systems, we define a joint MEM selection likelihood that integrates information from both the exposure-mediator and mediator-outcome pathways, yielding an adaptive error-allocation scheme under a fixed error budget. When the outcome model is nonlinear, we develop an adaptive extension of the measurement error kernel regression operator (MEKRO; White et al. (2017)), in which mediator-specific smoothing bandwidths play the same role as the error precisions in the linear criterion: oversmoothing an informative coordinate substantially worsens model fit, whereas oversmoothing an irrelevant coordinate has little effect. This formulation captures mediator relevance through both activity in the exposure–mediator pathway and contribution to the nonlinear outcome surface, allowing candidate mediators to be prioritized under nonlinear relationships such as quadratic effects, exposure–mediator interactions, and mediator–mediator interactions.

Our contributions are threefold. First, we introduce a new mediator selection principle in which a mediator’s relevance is defined by the sensitivity of the joint mediation system to controlled perturbations induced by artificially infusing measurement errors, rather than through direct penalization or testing of mediation coefficients. Second, for linear mediation models, we develop a joint MEM-based selection likelihood that integrates information from both the exposure-mediator and mediator-outcome pathways, yielding an adaptive error-allocation mechanism that is particularly useful for mediator selection when mediation signals are weak or imbalanced between pathways. Third, the kernel extension supports mediator selection beyond the linear outcome models assumed by existing methods, accommodating nonlinear and interactive mediator–outcome structure without requiring its functional form to be specified in advance. Together, these contributions extend mediator selection to settings that existing coefficient-based methods do not address.

The remainder of the article is organized as follows. Section 2 presents the proposed adaptive linear MEM-based selection procedure. Section 3 introduces our adaptive kernel MEM extension for nonlinear mediator selection. Section 4 reports simulation studies comparing the proposed methods with existing approaches. Section 5 presents an analysis of a DNA methylation dataset to prioritize candidate CpG mediators in the association between childhood trauma and adult cortisol stress reactivity. We conclude with a discussion in Section 6. Additional algorithmic details and supplementary simulations are provided in Appendix.

## 2 MEM-based Mediator Selection for Linear Mediation Models

### 2.1 Formulation and models

Let X, $M={\left(M_{1},\ldots ,M_{p}\right)}^{\prime }$, and Y denote a quantitative exposure, *p*-dimensional mediator vector, and a continuous outcome, respectively. The observed data consist of *n* independent and identically distributed (i.i.d.) copies of (X, $M^{\prime }$, Y), indexed by i=1,…,n with n>p+2. As a pre-processing step, all variables are centered and standardized to have mean 0 and variance 1.

We aim to identify candidate mediators that are relevant to both the exposure–mediator and mediator–outcome pathways, defined as follows. Let h(x,m)=E(Y∣X=x,M=m) denote the conditional mean of the outcome, and for $j\;=\;1,\ldots ,p,\;h_{-j}(x,m_{-j})\;=\;E(Y|X\;=\;x,M_{-j}\;=\;m_{-j})$, where $M_{-j}$ and $m_{-j}$ denote the corresponding vectors with the jth component removed. Let $m_{j}(x)\;=\;E(M_{j}|X\;=\;x)$. The corresponding exposure–mediator and mediator–outcome relevance sets are ${𝒮}_{XM}=\left\{j\;:E\left[{\left\{m_{j}(X)-E(M_{j})\right\}}^{2}\right]>0\right\}$ and ${𝒮}_{MY}=\left\{j\;:E\left[{\left\{h(X,M)-h_{-j}(X,M_{-j})\right\}}^{2}\right]>0\right\}$, respectively. The target mediator set is their intersection $𝒮={𝒮}_{XM}\cap {𝒮}_{MY}$, so a mediator is retained only if it is relevant to both pathways.

We first consider the following linear mediation system

(1a) $$M=\alpha X+\epsilon_{m},$$

(1b) $$Y=\beta_{x}X+\beta_{m}^{\prime }M+\epsilon_{y},$$

where $\alpha \;=\;{\left(\alpha_{1},\ldots ,\alpha_{p}\right)}^{\prime }$ contains the exposure–mediator coefficients, $\beta_{x}$ is the exposure coefficient in the outcome model, $\beta_{m}\;=\;{\left(\beta_{m1},\ldots ,\beta_{mp}\right)}^{’}$ contains the mediator–outcome coefficients. We assume that $\epsilon_{m}\;∼\;𝒩(0,\Sigma_{\text{resM}})$, where $\Sigma_{\text{resM}}$ allows for residual dependence among the mediators, and that $\epsilon_{y}∼𝒩(0,\sigma_{\text{resY}}^{2})$. We further assume that X, $\epsilon_{m}$, and $\epsilon_{y}$ are mutually independent. Provided that $\Sigma_{\text{resM}}$ is positive definite, the target set 𝒮 reduces to the coefficient-support representation

$$𝒮=\{j\;:\alpha_{j}\neq 0,\;\beta_{mj}\neq 0\},$$

as shown in Appendix A.1.

This coefficient-support characterization under (1a)-(1b) can be interpreted causally if the system is regarded as structural and the standard identification conditions hold: consistency, positivity, no unmeasured confounding of the exposure–outcome, exposure–mediator, and mediator–outcome relationships, and no exposure-induced mediator–outcome confounder (Imai et al. 2010; VanderWeele et al. 2014). When measured confounders are present, the corresponding conditional positivity is further assumed. Measured pre-exposure confounders can be accommodated by residualizing X, M, and Y with respect to them prior to selection of mediators (Jones et al. 2024). When the confounders enter additively and linearly, this procedure is equivalent to including them directly in both linear models (1a)-(1b), by the Frisch–Waugh–Lovell theorem; for the nonlinear outcome model in Section 3, it provides an approximate linear adjustment. We also assume that there are no directed causal relationships among the mediators, and conditional on X and the measured confounders, their residual dependence is represented by $\Sigma_{\text{resM}}$. This assumption is not testable from the observed data and is discussed in more details in Section 6. Under these conditions, mediators in 𝒮 with nonzero indirect effects $\alpha_{j}\beta_{mj}$ represent the active linear causal mediation pathways.

If, in addition, X is Gaussian, models (1) imply that (X, $M^{\prime }$, Y) is jointly Gaussian. Because all variables are standardized, the covariance matrix reduces to the correlation matrix. Using the reticular action model formulation (McArdle 2005), this covariance matrix can be written as

(2) $$\Sigma ≔\left(\begin{array}{ccc} 1 & \sigma_{mx}^{\prime } & \sigma_{xy}\\ \sigma_{mx} & \Sigma_{M} & \sigma_{my}\\ \sigma_{xy} & \sigma_{my}^{\prime } & 1 \end{array}\right)={\left(\begin{array}{ccc} 1 & & \\ -\alpha & I_{p} & \\ -\beta_{x} & -\beta_{m}^{\prime } & 1 \end{array}\right)}^{-1}\left(\begin{array}{ccc} 1 & & \\ & \Sigma_{\text{res}M} & \\ & & \sigma_{\text{res}Y}^{2} \end{array}\right){\left(\begin{array}{ccc} 1 & -\alpha^{\prime } & -\beta_{x}\\ & I_{p} & -\beta_{m}\\ & & 1 \end{array}\right)}^{-1}.$$

This joint covariance representation makes explicit how the exposure, mediators, and outcome are linked under the linear mediation system, and it provides the working model on which the measurement-error infusion procedure introduced next is built.

### 2.2 MEM-based mediator selection via measurement-error infusion

We now adapt the MEM principle to the linear mediation system (1), infusing measurement error into the candidate mediators in a controlled manner. When each mediator $M_{j}$, j=1,…,p was infused with an independent Gaussian measurement error with variance $\sigma_{uj}^{2}=1∕\lambda_{j}$ and subsequently rescaled to unit variance, the resulting error-infused mediator would be

$$M_{\text{err},j}={\left\{\lambda_{j}{\left(1+\lambda_{j}\right)}^{-1}\right\}}^{1∕2}M_{j}+{\left\{{\left(1+\lambda_{j}\right)}^{-1}\right\}}^{1∕2}R_{j},$$

where $\lambda_{j}>0$ is the precision of the infused error and $R_{j}\;\overset{\text{i.i.d.}}{∼}𝒩(0,1)$ independent of (X, $M^{\prime }$, Y). The rescaling retains $\text{Var}(M_{\text{err},j})=1$. The covariance of $M_{\text{err},j}$ with any random variable V that has a finite second moment would be attenuated, i.e. $\mathrm{Cov}(V,M_{\text{err},j})={\left\{\lambda_{j}{\left(1+\lambda_{j}\right)}^{-1}\right\}}^{1∕2}\mathrm{Cov}(V,M_{j})$. Smaller values of $\lambda_{j}$ correspond to larger measurement-error variances and stronger attenuation of the association between $M_{j}$ and the rest of the mediation system.

Let $\lambda ={\left(\lambda_{1},\ldots ,\lambda_{p}\right)}^{\prime }$ and ${𝒟}_{u}=\text{diag}({\left\{\lambda_{1}{\left(1+\lambda_{1}\right)}^{-1}\right\}}^{1∕2},\ldots ,{\left\{\lambda_{p}{\left(1+\lambda_{p}\right)}^{-1}\right\}}^{1∕2})$. The joint distribution of (X, $M_{\text{err}}^{\prime }$, Y) is Gaussian with the covariance matrix of

(3) $$\Sigma_{\text{MEM}}(\Sigma ,\lambda )=\left(\begin{array}{ccc} 1 & \sigma_{mx}^{\prime }{𝒟}_{u} & \sigma_{xy}\\ {𝒟}_{u}\sigma_{mx} & {𝒟}_{u}\Sigma_{M}{𝒟}_{u}+I_{p}-{𝒟}_{u}^{2} & {𝒟}_{u}\sigma_{my}\\ \sigma_{xy} & \sigma_{my}^{\prime }{𝒟}_{u} & 1 \end{array}\right),$$

where $\Sigma_{M}$, $\sigma_{mx}$, $\sigma_{my}$ and $\sigma_{xy}$ are the blocks of Σ defined in (2). Let $\mu_{Y|X,M_{\text{err}}}$, $\Sigma_{\text{MEM},Y|X,M_{\text{err}}}$, $\mu_{M_{\text{err}}|X}$, and $\Sigma_{\text{MEM},M_{\text{err}}|X}$ denote the conditional means and covariance matrices induced by $\Sigma_{\text{MEM}}(\Sigma ,\lambda )$ through standard Gaussian conditioning. Following Stefanski et al. (2014) and omitting an additive constant independent of λ, the joint MEM log-likelihood is

(4) $$\ell_{\text{MEM}}(\Sigma ,\lambda )=\sum_{i=1}^{n}\log\;f\;(Y_{i}|X_{i},M_{i};\mu_{Y|X,M_{\text{err}}},\Sigma_{\text{MEM},Y|X,M_{\text{err}}})\;\;+\sum_{i=1}^{n}\log\;f\left(M_{i}|X_{i};\mu_{M_{\text{err}}|X},\Sigma_{\text{MEM},M_{\text{err}}|X}\right),$$

where f(⋅) denotes the Gaussian density of the corresponding dimension. Although (4) is derived under the hypothetical error-infused model for ${\left(X,M_{\text{err}}^{\prime },Y\right)}^{\prime }$, it is evaluated at the observed data and used as a working criterion to quantify the degradation of the joint mediation system due to controlled measurement errors infused into the mediators (Stefanski et al. 2014). Replacing Σ by an empirical estimate, $\hat{\Sigma }$ in (4) gives the MEM selection log-likelihood

(5) $$\ell_{\hat{\text{SEL}}}(\lambda )=\ell_{\text{MEM}}(\hat{\Sigma },\lambda )=\ell_{\hat{\text{SEL}},Y|X,M}(\lambda )+\ell_{\hat{\text{SEL}},M|X}(\lambda ).$$

Notice that the sample covariance $\hat{\Sigma }$ of ($X,M^{\prime },Y$) should be positive definite, and thus we require n>p+2. This dimension requirement is a structural feature of the criterion rather than an incidental numerical issue, and it delimits the regime in which the linear MEM criterion operates: the number of candidate mediators carried into the joint model must remain below the sample size. In truly high-dimensional applications, the criterion is therefore applied to a reduced candidate set, obtained by an upstream screening step or by fitting random subsets of manageable size, as we do in Section 5.

### 2.3 Adaptive linear MEM-based mediator selection

To achieve selection, the original MEM method imposes the precision-budget constraint

$$\sum_{j=1}^{p}\lambda_{j}\leq \tau ,\;\lambda_{j}\geq 0,\;\tau >0,$$

which is equivalent to requiring the harmonic mean of the injected error variances to be at least $p\tau^{-1}$. The nonzero τ guarantees the infused errors and governs the total precision of the measurement-error available to the candidate mediators. Smaller values of τ leave less precision to distribute, inducing greater overall information degradation and hence stronger regularization. Although $1∕\lambda_{j}$ is interpretable as the artificial error variance only for $\lambda_{j}>0,\lambda_{j}=0$ means $M_{\text{err},j}=R_{j}$, so all the information carried by the mediator $M_{j}$ is attenuated and thus should be excluded.

To incorporate heterogeneous mediation strengths (Jones et al. 2024), we introduce an adaptively weighted precision budget, $\sum_{j=1}^{p}{\bar{w}}_{j}\lambda_{j}\leq \tau$, where

$$w_{j}={\left(|{\hat{\alpha }}_{j}{\hat{\beta }}_{mj}|+\epsilon \right)}^{-\gamma },\;{\bar{w}}_{j}=p\;w_{j}{\left(\sum_{k=1}^{p}w_{k}\right)}^{-1}.$$

Following the adaptive-LASSO construction (Zou 2006), ${\hat{\alpha }}_{j}$ is the ordinary least-squares slope from regressing $M_{j}$ on X, and ${\hat{\beta }}_{mj}$ is the jth mediator coefficient from the joint regression of Y on (X,M). Here γ>0 controls adaptivity and ϵ>0 prevents excessive weights when a preliminary estimate is near zero; we used γ = 1 and ϵ = 10^−6^ in all reported analyses. Because a larger value of $|{\hat{\alpha }}_{j}{\hat{\beta }}_{mj}|$ yields a smaller ${\bar{w}}_{j}$, retaining a given $\lambda_{j}$ consumes less of the precision budget, and thus promotes the selection of $M_{j}$.

The adaptive MEM mediator selection is then obtained by solving

(6) $$\max_{\lambda \geq 0}\;\ell_{\hat{\text{SEL}}}(\lambda )\;\text{s.t.}\;\sum_{j=1}^{p}{\bar{w}}_{j}\lambda_{j}\leq \tau ,\lambda_{j}\geq 0\;\text{for}\;j=1,\ldots ,p.$$

Because (6) allocates the precision budget according to importance in the *joint* mediation system, it may retain mediators that are active in only one of the exposure–mediator (X→M) or mediator–outcome ((X,M)→Y) pathways. We refer to these as “semi-mediators”. Stage 1 selection uses (6) to obtain this broader set. Stage 2 then restricts attention to the selected set from Stage 1 and optimizes the exposure–mediator component $\ell_{\hat{\text{SEL}},M|X}(\lambda )$ and the mediator–outcome component $\ell_{\hat{\text{SEL}},Y|X,M}(\lambda )$ from (5) separately, subject to the same adaptive weighted constraint. The final mediator set is the intersection of the two pathway-specific selections, so that retained variables contribute to both components of the mediation pathway.

The two stages follow similar optimization and tuning procedures, differing only in which component of the MEM selection likelihood is optimized and in the resulting BIC-type criterion. For fixed τ, we reparameterize the precision vector λ in terms of an unconstrained vector v as $\lambda_{j}(v)=\tau v_{j}^{2}∕(\sum_{k=1}^{p}{\bar{w}}_{k}v_{k}^{2})$, j=1,…,p. This transformation guarantees $\lambda_{j}(v)\geq 0$ for each j and $\sum_{j=1}^{p}{\bar{w}}_{j}\lambda_{j}(v)=\tau$. We use L-BFGS to optimize the MEM selection criterion under this full-budget parameterization. Selection is ultimately driven by the ranking of the ${\hat{\lambda }}_{j}$ rather than by their magnitudes, and we found this ranking to be stable across choices of τ in the linear MEM selection. Both stages therefore fix τ = 1 and run the optimization once.

Ranking ${\hat{\lambda }}_{j}$ in decreasing order produces nested candidate sets $S_{1}\subset S_{2}\subset \cdots \subset S_{p}$, where $S_{k}$ consists of the *k* highest-ranked mediators with the largest ${\hat{\lambda }}_{j}$. Each candidate set is evaluated using unpenalized refitting without error infusion. For Stage 1, we select S minimizing the plug-in BIC-type criterion

$${\text{BIC}}_{\text{plug},1}(S)=-2{\hat{\ell }}_{Y|X,M_{S}}+n\;\log\;\text{det}({\hat{\Sigma }}_{M|X}(S))+2\;\log(n)|S|,\;\;S\in \{S_{1},\ldots ,S_{p}\},$$

where ${\hat{\ell }}_{Y|X,M_{S}}$ is the Gaussian log-likelihood from the unpenalized outcome regression of Y on ($X,M_{S}$) and ∣S∣ denotes the cardinality of set S. Here, ${\hat{\Sigma }}_{M|X}(S)=n^{-1}\sum_{i=1}^{n}\{M_{i}-\hat{\alpha }(S)X_{i}\}{\left\{M_{i}-\hat{\alpha }(S)X_{i}\right\}}^{\prime }$, where $\hat{\alpha }(S)$ contains the marginal least-squares exposure coefficients for mediators in S and zeros elsewhere. The Stage 1 penalty counts two coefficients per selected mediator, one for each pathway; nuisance-parameter penalties common to all candidates are omitted. Stage 2 applies the same construction to one pathway at a time, refitting only that pathway and penalizing log(n)∣S∣ in its respective BIC-type plug-in criterion, since then a single coefficient per mediator is involved.

## 3 Adaptive Kernel MEM-based Mediator Selection

In practice, the outcome model may involve nonlinear exposure or mediator effects, exposure–mediator interactions, and interactions among mediators, none of which the linear specification (1) can adequately represent. Methods for mediator selection that accommodate such structures remain scarce: some existing nonlinear approaches target effect estimation (Loh et al. 2022) or gene-level testing (Fang et al. 2021) rather than the selection of multiple mediators. In this section, we extend the MEM principle to accommodate nonlinear mediator–outcome models, replacing the infused error precisions by mediator-specific inverse-bandwidth parameters in the outcome model, while retaining the linear exposure–mediator model (1a).

Specifically, we extend the linear outcome model (1b) to

(7a) $$Y=h(X,M)+\epsilon_{y},$$

where the conditional mean function h(x,m)=E(Y∣X=x,M=m) is left unspecified, and $E(\epsilon_{y}|X,M)=0$, allowing nonlinear main effects and interactions involving the exposure and mediators. The target remains the set 𝒮 of Section 2.1, with relevance in the mediator–outcome pathway now arising through h rather than through a coefficient. For causal interpretation, we assume the same identification conditions as in the linear setting.

### 3.1 Kernel MEM-based mediator selection

In kernel construction, increasing the bandwidth along a coordinate flattens the effects from that variable: oversmoothing an informative coordinate substantially worsens model fit, whereas oversmoothing an irrelevant coordinate has little effect. This is the principle underlying the measurement-error kernel regression operator (MEKRO) of White et al. (2017) for variable selection.

Let $Z={\left(X,M^{\prime }\right)}^{\prime }$ and $z={\left(x,m^{\prime }\right)}^{\prime }$. For $\lambda ={\left(\lambda_{1},\ldots ,\lambda_{p}\right)}^{\prime }$, with $\lambda_{j}\geq 0$, define

(8) $$K_{\lambda }(z,z^{\prime })=K_{X}(x,x^{\prime })\prod_{j=1}^{p}\text{exp}\left\{-\frac{\lambda_{j}^{2}}{2}{\left(m_{j}-m_{j}^{\prime }\right)}^{2}\right\}.$$

where $K_{X}(x,x^{\prime })$ is a Gaussian kernel for the exposure coordinate, whose bandwidth is selected from the data and then held fixed during mediator selection. For example, we choose the bandwidth for the exposure by minimizing the leave-one-out squared error using an unpenalized product-kernel Nadaraya–Watson regression of Y on (X, M) in all numerical studies. The fixed $K_{X}(x,x^{\prime })$ ensures that the exposure always remains in the outcome model. Smaller values of $\lambda_{j}$ correspond to larger effective bandwidths and hence stronger smoothing along mediator $M_{j}$. Thus, a mediator that can tolerate substantial smoothing without appreciable loss of fit is regarded as weak or uninformative. Adopting the MEKRO framework, we select the mediators for (X,M)→Y pathway by solving

(9) $$\min_{\lambda \geq 0}\sum_{i=1}^{n}{\left\{Y_{i}-\hat{h}(Z_{i};\lambda )\right\}}^{2}\;\text{s.t.}\;\sum_{j=1}^{p}w_{j}\lambda_{j}\leq \tau ,\;\lambda_{j}\geq 0,\;\;j=1,\ldots ,p,$$

where $\hat{h}(z;\lambda )\;=\;{\left\{\sum_{i=1}^{n}K_{\lambda }(z,Z_{i})\right\}}^{-1}\sum_{i=1}^{n}Y_{i}K_{\lambda }(z,Z_{i})$, τ>0 is the mediator inverse-bandwidth budget, and $w_{j}$ are the adaptive weights defined below. When $\lambda_{j}=0$, the corresponding kernel factor equals one, so the fitted smoother does not depend on $M_{j}$.

For each budget value τ, we solve (9) and prune the mediator coordinates by sequentially dropping the least important one while the change in the objective function value stays within a fixed tolerance. The Nadaraya–Watson optimization and pruning therefore produce a candidate mediator subset for each τ. To evaluate these subsets, we fit a separate kernel ridge regression (KRR) of Y on X and the mediators selected at each τ. We then select τ over a prespecified grid by minimizing the generalized cross-validation (GCV) score of the corresponding KRR fit. Let ${\hat{𝒮}}_{MY}$ denote the resulting selected set for the mediator–outcome pathway. The final selection is ${\hat{𝒮}}_{XM}\cap {\hat{𝒮}}_{MY}$, where ${\hat{𝒮}}_{XM}$ is obtained using the approach in Section 2.3. Computational details are given in Appendix Section A.2.

### 3.2 Adaptive weights based on in-sample $R^{2}$ importance

Because the nonlinear outcome model lacks a closed-form analogue of the linear proxy $\alpha_{j}\beta_{mj}$, we construct adaptive weights from pathway-specific $R^{2}$ measures. For each mediator $M_{j}$, let ${\hat{R}}_{M_{j}}^{2}$ denote the in-sample coefficient of determination from the least-squares regression of $M_{j}$ on X. For the outcome pathway, kernel ridge regression with Gaussian product kernel (Saunders et al. 1998) is fitted to (X,M) and to ($X,M_{-j}$), yielding ${\hat{R}}_{\text{full}}^{2}$ and ${\hat{R}}_{-M_{j}}^{2}$, respectively. We define $\Delta {\hat{R}}_{M_{j}}^{2}={\hat{R}}_{\text{full}}^{2}-{\hat{R}}_{-M_{j}}^{2}$ and then the adaptive weights as

$$w_{j}={\left[\left({\hat{R}}_{M_{j}}^{2}+\epsilon \right)\left(\max\{\Delta {\hat{R}}_{M_{j}}^{2},0\}+\epsilon \right)\right]}^{-\gamma },\;{\tilde{w}}_{j}=p\;w_{j}{\left(\sum_{k=1}^{p}w_{k}\right)}^{-1},$$

with ϵ>0 and γ>0 similarly defined as in Section 2.3. Larger weights encourage more smoothing for the weaker candidate mediators so they can be selected out easily. Details about computing weights are given in Appendix Section A.2.

## 4 Simulations

### 4.1 Settings

We evaluated the proposed methods under four scenarios: one linear mediation setting (**S1**) and three settings (**S2**–**S4**) with nonlinear mediator-outcome relationships. Throughout, the term “true mediator” is used as shorthand for a variable in the prespecified two-pathway active set 𝒮, that is, a variable active in both the exposure-mediator and mediator-outcome pathways. Setting **S1** evaluates selection under a correctly specified linear outcome model, including the separation of true mediators from semi-mediators. Settings **S2**–**S4** assess selection when outcome relevance arises through nonlinear effects or interactions. Detailed settings are specified as follows.

We considered sample sizes n∈{100,200} and *p* = 22 mediators, where the choice of p approximated n∕log(n) for n = 100, a dimension commonly retained after screening by HIMA and HDMA (Zhang et al. 2016; Gao et al. 2019). We generated X∼N(0,1) and $M\;=\;\alpha X+\epsilon_{m}$, where $\epsilon_{m}$ denoted a p×1 vector of Gaussian mediator residuals that were independent of X, had mean zero and marginal variance of 1, and with a correlation matrix of independent or AR(1) correlation ${\left\{\Sigma_{\text{res}M}\right\}}_{jk}\;=\;0.5^{|j-k|}$. Under the AR(1) structure, we further randomly permuted the mediator indices in each replication before assigning the active mediators, so that each replication drew a different correlation configuration among the mediators. After X and M were generated, the residual variance of the outcome was calibrated as $\sigma_{\text{res}Y}^{2}=r∕(1-r)\text{Var}\{h(X,M)\}$ to achieve the target unexplained residual variance proportion r∈{0.75,0.50}, and Y was generated as $h(X,M)+\epsilon_{y}$ where $\epsilon_{y}$ was generated independently of (X,M) from $N(0,\sigma_{\text{res}Y}^{2})$. Only after this full sequence were X, M, and Y each centered and rescaled to unit variance, so the residual-share calibration reflected the pre-standardization scale of the model. Because the two-pathway target set 𝒮 is invariant to linear standardization, this operation does not affect which mediators are active.

- **S1 (Linear).** We set $\alpha \;=\;{\left(0.55,0.25,0.15,0.25,0.25,0.55,0,0,0,\ldots ,0\right)}^{\prime }$ and generated the outcome $Y\;=\;0.38X+0.4M_{1}+0.35M_{2}+0.2M_{3}+0.4M_{4}+0.35M_{7}+0.40M_{8}+\epsilon_{y}$. This value of $\beta_{x}\;=\;0.38$ yielded a variance ratio $\text{Var}(\beta_{x}X)∕\text{Var}(\beta_{x}X+\beta_{m}^{\prime }M)$ at 0.1 under independent mediator residuals. This configuration contained four true mediators, $M_{1}-M_{4}$, with varying mediation strengths; two exposure-only semi-mediators, $M_{5}$ and $M_{6}$, with $\alpha_{j}\neq 0,\beta_{mj}=0$; two outcome-only semi-mediators, $M_{7}$ and $M_{8}$, with $\alpha =0,\beta_{m}\neq 0$, and the null mediators, $M_{9}-M_{22}$. A linear setting featuring random model coefficients and a larger mediator dimension (p=50) is included in Appendix Section B.
- **S2–S4 (Nonlinear).** We set $\alpha ={\left(1∕3,1∕3,1∕3,0,\ldots ,0\right)}^{\prime }$ so that X explained 10% of $\text{Var}(M_{j})$ for $M_{1}-M_{3}$, while the remaining mediators had inactive exposure–mediator pathways.
  - **S2 (Quadratic):** $Y=\beta_{x}X+2M_{1}^{2}+M_{2}+M_{4}+\epsilon_{y}$.
  - **S3 (Exposure-mediator interaction)**: $Y=0.25X-0.25M_{1}+XM_{1}+M_{2}+M_{4}+\epsilon_{y}$.
  - **S4 (Mediator–mediator interaction)**: $Y=\beta_{x}X+M_{2}+M_{4}+M_{1}M_{4}+\epsilon_{y}$.

In settings **S2** and **S4**, $\beta_{x}$ was calibrated to satisfy the variance ratio $\text{Var}(\beta_{x}X)∕\text{Var}\{h(X,M)\}=0.1$, recomputed and recalibrated in each replication using the sample variances and under each permuted mediator-correlation configuration. Under **S2–S4**, $M_{1}$ and $M_{2}$ were the true mediators. Specifically, $M_{1}$ contributed to the outcome either nonlinearly or through an interaction term, whereas $M_{2}$ had a linear effect in all settings. Mediator $M_{3}$ was an exposure-only semi-mediator as it had α≠0 but was absent from h(X,M), whereas $M_{4}$ was an outcome-only semi-mediator since it entered h(X,M) but had α=0. The remaining $M_{5}-M_{22}$ were null mediators.

We conducted 100 Monte Carlo replications under each setting and compared the proposed linear and kernel MEM-based selection methods with HIMA, HDMA, and RegSEM. For the linear setting (**S1**), additional comparisons with Pathway LASSO and BSLMM are reported in Appendix Section C. The R package hdmed was used to implement HIMA, HDMA, Pathway LASSO, and BSLMM. For the nonlinear settings **S2**–**S4**, we additionally evaluated a KRR-importance comparator, where the exposure–mediator pathway was selected in the same way as in kernel MEM selection and the mediator–outcome pathway was selected by the KRR importance measure. The KRR importance comparator provides a further benchmark for determining whether kernel MEM offers selection advantages beyond flexible nonlinear prediction and pathway screening. Specifically, for the mediator–outcome pathway, observations were first partitioned into five folds. Within each fold, a kernel ridge regression of Y on X and all p mediators was fitted on the training portion, and its mean squared error was evaluated on the held-out portion. The importance of mediator $M_{j}$ in a given fold was quantified as the increase in held-out mean squared error of the fitted KRR model when the values of $M_{j}$ in that fold were randomly permuted, averaged over 20 permutations. This quantity was then averaged across the five folds to obtain the mean importance of $M_{j}$. Mediators with positive mean importance were ranked in decreasing order of importance, yielding a nested sequence of top-k mediator sets. For each k in this sequence, the corresponding model was evaluated by cross-validation on the same five folds. We chose the smallest k whose mean held-out error fell within one standard error of the minimum mean held-out error along this path. The final selected mediator set was the intersection of the exposure–mediator and mediator–outcome selections.

### 4.2 Simulation Results

Table 1 summarizes the selection probabilities of mediators under setting **S1**. Overall, linear MEM was competitive with the other procedures, with more satisfactory performance depending on the mediator and residual correlation structure. RegSEM selected true mediators more frequently in several configurations but also more frequently retained semi-mediators. When the mediator residuals were independent (ρ = 0), HDMA generally showed higher selection probabilities, particularly at the larger sample size of n = 200. In contrast, the advantage of linear MEM-based selection was more pronounced under correlated mediator residuals (ρ = 0.5). This improvement could be attributed to the joint modeling of multiple mediators in the MEM framework, together with the adaptive weighting strategy, which enhanced the selection of the strongest mediator $M_{1}$ while maintaining comparable performance for $M_{2}$ except with 50% outcome residual variance combined and n = 200. For the weaker or more imbalanced mediators $M_{3}$ and $M_{4}$, differences among the methods were less consistent, but linear MEM remained competitive with HDMA under correlated mediator residuals, particularly at the smaller sample size.

Selection of the completely null mediators $M_{9}-M_{22}$ remained uniformly low for all methods. Differences were more apparent for the semi-mediators, with linear MEM and RegSEM more frequently retaining the exposure-only semi-mediators $M_{5}$ and $M_{6}$ than HIMA and HDMA. These results indicate that distinguishing exposure-only semi-mediators from true mediators remained more difficult than excluding completely null mediators. Selection of the outcome-only semi-mediators $M_{7}$ and $M_{8}$ remained comparatively low.

We also conducted a sensitivity analysis on the performance of the linear MEM procedure under the skewed-normal and Gaussian-mixture designs and reported results in Appendix Section D. Despite departures from the Gaussian assumption, the true-mediator selection probability remained stable, differing only slightly from its value under Gaussian errors across all examined configurations. Selection of null mediators remained low under both Gaussian and non-Gaussian sampling. Overall, the linear MEM method provides a principled new approach that is competitive with leading linear alternatives, performs particularly well under correlated mediators and pathway imbalance, but could be vulnerable to exposure-only semi-mediators.

Tables 2 to 4 report selection probabilities of candidate mediators under settings **S2**–**S4**, respectively. In **S2** (Table 2), where $M_{1}$ entered the outcome model quadratically, the kernel MEM showed substantially higher selection probabilities for $M_{1}$ than the linear methods across all configurations. At n = 100, ρ = 0, and a 50% outcome residual variance, kernel MEM selected $M_{1}$ with probability 0.85, compared with 0.77 for KRR importance, 0.44 for linear MEM, and 0.26 for HDMA. The advantage over the linear methods remained pronounced at n = 200, where the corresponding probabilities were 0.99, 1.00, 0.39, and 0.23, respectively. Under the weaker signal–to–noise ratio (i.e. 75% outcome residual) setting, kernel MEM also showed a clearer advantage over KRR importance when n = 100, with probabilities of 0.73 versus 0.31 for ρ = 0 and 0.79 versus 0.39 for ρ = 0.5. For the linearly contributing mediator $M_{2}$, kernel MEM performed comparably to linear MEM; for example, at ρ=0 and a 50% residual fraction, the selection probabilities were 0.25 versus 0.24 at n = 100 and 0.32 versus 0.31 at n = 200.

Similar patterns were observed in the two interaction settings. In **S3** (Table 3), where $M_{1}$ contributed through an $X\times M_{1}$ interaction, kernel MEM selected $M_{1}$ with probability 0.75 at *n* = 100, ρ = 0, and a 50% outcome residual variance, compared with 0.59 for KRR importance, 0.27 for linear MEM, and 0.20 for HDMA. In **S4** (Table 4), where $M_{1}$ contributed through the $M_{1}M_{4}$ interaction, the corresponding probabilities were 0.47, 0.35, 0.06, and 0.11. In both settings, kernel MEM maintained a clear advantage over the linear methods with increasing sample size and under correlated mediator residuals. The comparison with the KRR importance comparator depended on outcome signal strength: at the 75% residual fraction, kernel MEM retained the advantage in both settings, with $M_{1}$ selection probabilities of 0.96 versus 0.53 in **S3** and 0.51 versus 0.31 in **S4** at *n* = 200 and ρ = 0; at the 50% residual fraction, the two methods converged with increasing sample size in **S3**, while KRR importance exceeded kernel MEM at *n* = 200 in **S4**. Selection of the linearly contributing mediator $M_{2}$ remained generally high, particularly at the larger sample size.

Across **S2**–**S4**, selection of the completely null mediators $M_{5}-M_{22}$ remained uniformly low. For kernel MEM, false selection was concentrated primarily on $M_{3}$, the exposure-only semi-mediator. Selection of the outcome-only semi-mediator $M_{4}$ remained low in **S2** and **S3**, but increased in **S4** for KRR importance, reaching 0.21 and 0.39 at *n* = 200 with a 50% residual fraction under ρ = 0 and ρ = 0.5, respectively. This pattern was consistent with the stronger outcome-side contribution of $M_{4}$ in **S4**, where it entered through both its main effect and the $M_{1}M_{4}$ interaction. Overall, kernel MEM improved detection of mediators with nonlinear or interaction-driven outcome contributions relative to the linear methods, with its advantage over KRR importance most evident under weaker outcome signals.

## 5 Data Application

Adverse childhood experiences have been consistently associated with a wide range of adult mental health outcomes, and epigenetic alterations have been hypothesized to be potential mediators of these long-term effects (McKay et al. 2021). We applied the proposed MEM-based selection methods to a genome-wide epigenetic dataset (ArrayExpress accession: E-GEOD-77445) collected by Houtepen et al. (2016). The study included 85 healthy individuals and investigated how childhood trauma (exposure) affects adult cortisol stress reactivity (outcome) through DNA methylation measured at 385, 882 CpG sites. This dataset has been analyzed previously using a variety of mediation and variable-selection methods, with substantial variability in the reported CpG loci (Table 5). For example, Houtepen et al. (2016) reported three loci and van Kesteren and Oberski (2019) reported five different loci. Guo et al. (2022) considered these eight loci jointly and selected three additional loci, noting that several previously reported loci were no longer significant after joint modeling. Separately, Schaid and Sinnwell (2020) applied RegSEM and identified four loci, with one overlapping with van Kesteren and Oberski (2019).

### 5.1 Analytic pre-processing

We first performed a screening to retain the top 200 candidate mediators ranked by the marginal mediation score defined as $|{\hat{\alpha }}_{j}{\hat{\beta }}_{j}|$, where ${\hat{\alpha }}_{j}$ was obtained by regressing $M_{j}$ on X, and ${\hat{\beta }}_{j}$ was obtained from the regression of Y on (X, $M_{j}$), both adjusting for age, sex, and estimated proportions of B cells, CD4 T cells, CD8 T cells, monocytes, granulocytes, and natural killer cells (Jones et al. 2024). We supplemented this set with previously reported loci (listed in Table 5), resulting in a candidate set of 213 CpG sites for analysis. To adjust for the demographic and clinical confounders in mediator selection procedures, we residualized X, each $M_{j}$, and Y with respect to the same set of covariates and then standardized the adjusted variables. To mitigate the impact of mediator correlation and improve computational stability, we analyzed randomly sampled subsets of 20 candidate mediators at a time using either the linear or kernel MEM-based selection method. We repeated this procedure 1, 065 times, so that each CpG site appeared in approximately 100 subsets (median=99 and range = (75,113)). For mediator $M_{j}$, we calculated its conditional selection frequency in all co-mediator subsets containing $M_{j}$ and retained loci with their conditional selection frequency above 80%; this pragmatic threshold retained no more than 20 mediators for the final joint fit for either MEM method. Finally, each method was fitted to its refined set for final joint selection. The resulting selections are summarized in Table 5.

### 5.2 Analysis Results

The linear MEM-based method pre-selected 10 loci with selection frequencies above 80% and identified eight final loci (Table 5), three of which overlapped with loci reported in the four prior studies summarized in Table 5. Specifically, cg16657538 (annotated to *ZSCAN30*) was previously reported by van Kesteren and Oberski (2019). cg19230917 (*RAB5IF*) was reported by Guo et al. (2022) and has been linked to endocytic processes that mediate cellular internalization of extracellular substances. The locus cg01644731 (*CTDSP2*) overlapped with Schaid and Sinnwell (2020) and has been related to stress responses and psychiatric disorders. In addition, cg25448067 was reported as part of a jointly mediating group in Wang et al. (2025); cg03025830 (*FGF17*) has shown neuroprotective effects and improved cognitive function in aging mouse models (Oberholzer et al. 2024); cg03643137 (*CNOT1*) has been associated with neurodevelopmental delay (Vissers et al. 2020); cg07343612 (*PIGQ*) encodes a subunit of the GPI-N-acetylglucosaminyltransferase complex that initiates glycosylphosphatidylinositol anchor biosynthesis; biallelic pathogenic variants cause early infantile epileptic encephalopathy and have been implicated in congenital hypotonia and developmental delay (Johnstone et al. 2020); and cg16503628 (*FGF12*) relates to nervous system disorders and neuronal excitability (Biadun et al. 2024).

The kernel MEM-based selection method pre-selected 9 loci and retained three in the final set (Table 5). Among these, cg25626453 (*PRRC2A*) was previously reported by both van Kesteren and Oberski (2019) and Schaid and Sinnwell (2020), and cg07343612 (*PIGQ*), described above, was also selected by the linear MEM-based method. The third selected locus, cg27103436, is intergenic.

## 6 Discussion

We developed a measurement error modeling (MEM)-based framework for selecting mediators in linear and nonlinear mediation systems. In the linear procedure, artificially infused measurement error perturbs the joint exposure–mediator and mediator–outcome system. The kernel extension implements a similar principle through mediator-specific inverse bandwidths while holding the exposure kernel fixed, thereby retaining the exposure as an unpenalized conditioning variable. The simulation results showed that linear MEM was competitive with the leading linear alternatives and that kernel-based MEM selection outperformed others for the nonlinear mediators. Kernel-based MEM also selected the nonlinear or interaction-driven mediator more frequently than the generic KRR importance comparator in most reported configurations, although the difference narrowed under the stronger signals in **S2** and **S3**. In the DNA methylation application, linear and kernel MEM identified eight and three final loci, respectively, including loci reported in previous studies.

Mechanistically, MEM-based selection operates by infusing artificial measurement error into candidate mediators and evaluating a controlled degradation of the mediation system. This perturbation perspective is conceptually related to interventional indirect effects (VanderWeele et al. 2014; Loh et al. 2022), which evaluate changes in the expected outcome under interventions that shift mediator distributions between those associated with different exposure levels. This connection provides additional motivation for applying MEM in multiple-mediator analysis. By modeling the mediators jointly, our procedure accommodates statistical dependence among mediators without requiring a specified causal ordering for the screening step. Accordingly, MEM-based selection may provide a useful prioritization step before estimation of interventional indirect effects. Heuristically, when perturbations on a mediator produce only limited changes in the outcome model, that mediator may be less likely to be retained by MEM-based selection.

Several limitations remain. First, the procedures prioritize variables with empirical support in both pathways rather than estimate a causal mediation effect. Second, the framework performs selection rather than inference; post-selection effect estimation, uncertainty quantification, and sensitivity analysis require separate procedures. Third, the full linear criterion requires a nonsingular empirical covariance matrix and is therefore restricted to moderate-dimensional problems after screening. The application also used literature augmentation and a pragmatic stability threshold; sensitivity to the screen size, subset size, selection-frequency threshold, and random subset sampling remains to be assessed. Fourth, residualizing covariates before fitting the nonlinear outcome model is a computational approximation; treating required adjustment variables as fixed, unpenalized kernel inputs would better preserve nonlinear adjustment. Finally, kernel MEM does not recover the functional form of a selected mediator’s effect and may have limited sensitivity to weak interactions. Extending MEM to simultaneous selection and structure learning is an important direction (Wu and Stefanski 2015).

## 7 Conclusions

The adaptive linear and kernel MEM procedures prioritize candidate mediators by allocating a fixed information-degradation budget across mediators. The linear procedure uses a joint Gaussian working criterion, whereas the kernel procedure accommodates nonlinear and interactive mediator–outcome relationships while keeping the exposure unpenalized. Both methods are intended for use in moderate-dimensional settings and identify candidates with evidence in both pathways. The simulations characterize their selection performance, and the DNA methylation analysis illustrates their application to high-dimensional molecular data. Effect estimation, post-selection inference, and biological validation remain separate tasks.

## Supplementary Material

**Supplementary information.** Additional algorithmic details and supplementary simulations are provided in the Appendix.

## Figures and Tables

**Table 1:** Selection probabilities of candidate mediators under Linear Setting **S1**. Selection probabilities of candidate mediators across four methods (Linear MEM, HIMA, HDMA, and RegSEM) under linear scenario **S1**, for varying correlation among mediators (ρ), percentage of outcome variance unexplained by the exposure and true mediators ($Y_{\text{residual}}\%$), and sample size (*n*). $M_{1}-M_{4}$ are true mediators, with $\alpha_{j}\neq 0$ and $\beta_{mj}\neq 0$; $M_{5}$ and $M_{6}$ are exposure-only semi-mediators, with $\alpha_{j}\neq 0$ and $\beta_{mj}=0$; $M_{7}$ and $M_{8}$ are outcome-only semi-mediators, with $\alpha_{j}=0$ and $\beta_{mj}\neq 0$; and “Others” refers to the null mediators $M_{9}-M_{22}$, which have no active causal pathway.

ρ	$Y_{\text{residual}}\%$	*n*	Method	$M_{1}$	$M_{2}$	$M_{3}$	$M_{4}$	$M_{5}$	$M_{6}$	$M_{7}$	$M_{8}$	Others
0.0	75%	100	Linear MEM	0.57	0.24	0.05	0.26	0.07	0.06	0.03	0.01	0.00
			HIMA	0.34	0.20	0.04	0.16	0.04	0.00	0.02	0.02	0.00
			HDMA	0.63	0.39	0.07	0.38	0.09	0.04	0.02	0.03	0.00
			RegSEM	0.48	0.24	0.05	0.20	0.07	0.00	0.02	0.03	0.00
		200	Linear MEM	0.81	0.44	0.06	0.53	0.05	0.06	0.04	0.03	0.00
			HIMA	0.58	0.39	0.06	0.46	0.04	0.04	0.07	0.02	0.00
			HDMA	0.89	0.64	0.17	0.82	0.07	0.03	0.05	0.02	0.00
			RegSEM	0.83	0.60	0.18	0.68	0.13	0.06	0.06	0.05	0.01
	50%	100	Linear MEM	0.82	0.50	0.14	0.58	0.10	0.07	0.08	0.08	0.00
			HIMA	0.77	0.50	0.10	0.52	0.05	0.00	0.03	0.07	0.00
			HDMA	0.90	0.58	0.16	0.59	0.08	0.02	0.03	0.06	0.00
			RegSEM	0.93	0.54	0.13	0.52	0.12	0.10	0.02	0.04	0.01
		200	Linear MEM	0.99	0.83	0.22	0.90	0.04	0.07	0.10	0.04	0.00
			HIMA	0.98	0.86	0.24	0.88	0.04	0.00	0.08	0.04	0.00
			HDMA	0.99	0.90	0.33	0.93	0.05	0.03	0.08	0.04	0.00
			RegSEM	1.00	0.83	0.30	0.86	0.23	0.14	0.04	0.04	0.01
0.5	75%	100	Linear MEM	0.59	0.25	0.03	0.24	0.04	0.08	0.01	0.05	0.01
			HIMA	0.33	0.17	0.02	0.20	0.02	0.06	0.02	0.01	0.00
			HDMA	0.42	0.21	0.04	0.26	0.02	0.05	0.02	0.01	0.00
			RegSEM	0.47	0.26	0.04	0.29	0.05	0.12	0.06	0.03	0.01
		200	Linear MEM	0.78	0.43	0.09	0.56	0.11	0.13	0.04	0.03	0.00
			HIMA	0.58	0.36	0.11	0.50	0.04	0.02	0.01	0.00	0.00
			HDMA	0.66	0.38	0.09	0.54	0.04	0.05	0.01	0.01	0.00
			RegSEM	0.76	0.58	0.21	0.73	0.16	0.15	0.06	0.09	0.02
	50%	100	Linear MEM	0.82	0.47	0.07	0.49	0.02	0.09	0.08	0.10	0.01
			HIMA	0.65	0.35	0.07	0.43	0.04	0.07	0.02	0.02	0.00
			HDMA	0.79	0.38	0.05	0.46	0.02	0.06	0.03	0.02	0.00
			RegSEM	0.93	0.50	0.14	0.61	0.11	0.21	0.15	0.06	0.02
		200	Linear MEM	0.97	0.78	0.25	0.85	0.08	0.15	0.10	0.11	0.00
			HIMA	0.90	0.82	0.28	0.86	0.05	0.04	0.04	0.03	0.00
			HDMA	0.97	0.84	0.27	0.88	0.05	0.07	0.05	0.03	0.00
			RegSEM	0.99	0.82	0.41	0.87	0.17	0.26	0.09	0.12	0.02

**Table 2:** Selection probabilities of candidate mediators under Quadratic Setting **S2**. Selection probabilities of candidate mediators across six methods (Kernel MEM, KRR importance, Linear MEM, HIMA, HDMA, and RegSEM) under scenario S2 with a quadratic $M_{1}$ effect, for varying correlation among mediators (ρ), percentage of outcome variance unexplained by the exposure and true mediators ($Y_{\text{residual}}\%$), and sample size (*n*). $M_{1}$ and $M_{2}$ are true mediators; $M_{3}$ is an exposure-only semi-mediator; $M_{4}$ is an outcome-only semi-mediator; and “Others” refers to the null mediators $M_{5}-M_{22}$, which have no active causal pathway.

ρ	$Y_{\text{residual}}\%$	Method	*n* = 100					*n* = 200				
			$M_{1}$	$M_{2}$	$M_{3}$	$M_{4}$	Others	$M_{1}$	$M_{2}$	$M_{3}$	$M_{4}$	Others
0.0	75%	Kernel MEM	0.73	0.30	0.24	0.03	0.01	0.97	0.32	0.28	0.00	0.00
		KRR importance	0.31	0.07	0.03	0.02	0.00	0.81	0.07	0.06	0.00	0.00
		Linear MEM	0.34	0.27	0.15	0.01	0.01	0.41	0.28	0.21	0.01	0.00
		HIMA	0.05	0.04	0.02	0.00	0.00	0.07	0.04	0.01	0.00	0.00
		HDMA	0.18	0.10	0.04	0.01	0.00	0.15	0.17	0.09	0.00	0.00
		RegSEM	0.04	0.02	0.01	0.00	0.00	0.11	0.07	0.05	0.00	0.00
	50%	Kernel MEM	0.85	0.25	0.09	0.00	0.00	0.99	0.32	0.19	0.00	0.00
		KRR importance	0.77	0.12	0.03	0.01	0.01	1.00	0.03	0.00	0.00	0.00
		Linear MEM	0.44	0.24	0.14	0.01	0.01	0.39	0.31	0.25	0.01	0.00
		HIMA	0.12	0.06	0.02	0.01	0.00	0.14	0.06	0.02	0.00	0.00
		HDMA	0.26	0.21	0.07	0.01	0.00	0.23	0.29	0.09	0.00	0.01
		RegSEM	0.09	0.01	0.00	0.00	0.00	0.18	0.20	0.03	0.00	0.00
0.5	75%	Kernel MEM	0.79	0.24	0.20	0.02	0.01	1.00	0.34	0.22	0.01	0.00
		KRR importance	0.39	0.07	0.05	0.03	0.01	0.94	0.03	0.05	0.01	0.01
		Linear MEM	0.42	0.19	0.12	0.01	0.01	0.30	0.28	0.22	0.03	0.01
		HIMA	0.05	0.03	0.02	0.00	0.00	0.10	0.04	0.01	0.00	0.00
		HDMA	0.11	0.10	0.05	0.00	0.00	0.13	0.08	0.04	0.00	0.00
		RegSEM	0.01	0.04	0.02	0.00	0.00	0.08	0.08	0.00	0.00	0.00
	50%	Kernel MEM	0.91	0.17	0.04	0.01	0.00	1.00	0.34	0.12	0.00	0.00
		KRR importance	0.85	0.07	0.02	0.01	0.01	0.99	0.01	0.00	0.00	0.00
		Linear MEM	0.44	0.17	0.16	0.00	0.01	0.39	0.27	0.20	0.03	0.01
		HIMA	0.14	0.06	0.02	0.00	0.00	0.16	0.15	0.02	0.00	0.00
		HDMA	0.17	0.12	0.05	0.00	0.00	0.19	0.16	0.02	0.00	0.00
		RegSEM	0.09	0.05	0.02	0.00	0.00	0.13	0.17	0.03	0.00	0.00

**Table 3:** Selection probabilities of candidate mediators under Exposure–Mediator Interaction Setting **S3**. Selection probabilities of candidate mediators across six methods (Kernel MEM, KRR importance, Linear MEM, HIMA, HDMA, and RegSEM) under scenario S3 with an interaction term between $M_{1}$ and the exposure, for varying correlation among mediators (ρ), percentage of outcome variance unexplained by the exposure and true mediators ($Y_{\text{residual}}\%$), and sample size (*n*). $M_{1}$ and $M_{2}$ are true mediators; $M_{3}$ is an exposure-only semi-mediator; $M_{4}$ is an outcome-only semi-mediator; and “Others” refers to the null mediators $M_{5}-M_{22}$, which have no active causal pathway.

ρ	$Y_{\text{residual}}\%$	Method	*n* = 100					*n* = 200				
			$M_{1}$	$M_{2}$	$M_{3}$	$M_{4}$	Others	$M_{1}$	$M_{2}$	$M_{3}$	$M_{4}$	Others
0.0	75%	Kernel MEM	0.58	0.87	0.25	0.03	0.01	0.96	0.97	0.27	0.00	0.00
		KRR importance	0.21	0.63	0.06	0.02	0.01	0.53	0.92	0.08	0.04	0.01
		Linear MEM	0.14	0.91	0.05	0.02	0.00	0.09	0.98	0.05	0.01	0.00
		HIMA	0.09	0.73	0.04	0.00	0.00	0.08	0.96	0.01	0.00	0.00
		HDMA	0.10	0.84	0.04	0.03	0.00	0.16	0.97	0.05	0.02	0.00
		RegSEM	0.06	0.60	0.02	0.00	0.00	0.18	0.93	0.05	0.00	0.00
	50%	Kernel MEM	0.75	0.99	0.14	0.05	0.01	0.99	0.98	0.19	0.00	0.00
		KRR importance	0.59	0.94	0.04	0.06	0.01	0.99	0.99	0.04	0.04	0.00
		Linear MEM	0.27	0.97	0.03	0.06	0.00	0.38	0.97	0.03	0.01	0.00
		HIMA	0.19	0.94	0.04	0.05	0.00	0.24	0.97	0.01	0.03	0.00
		HDMA	0.20	0.94	0.04	0.05	0.00	0.39	0.97	0.04	0.03	0.01
		RegSEM	0.19	0.75	0.02	0.03	0.00	0.39	0.94	0.09	0.04	0.00
0.5	75%	Kernel MEM	0.63	0.76	0.22	0.02	0.01	0.96	0.99	0.16	0.01	0.00
		KRR importance	0.20	0.50	0.06	0.02	0.01	0.46	0.93	0.03	0.04	0.01
		Linear MEM	0.11	0.82	0.06	0.01	0.00	0.17	0.99	0.02	0.04	0.00
		HIMA	0.10	0.70	0.06	0.01	0.00	0.13	0.97	0.02	0.01	0.00
		HDMA	0.09	0.76	0.04	0.02	0.00	0.12	0.98	0.04	0.01	0.00
		RegSEM	0.06	0.70	0.05	0.01	0.01	0.13	0.97	0.03	0.02	0.00
	50%	Kernel MEM	0.82	0.95	0.12	0.02	0.00	1.00	1.00	0.13	0.02	0.00
		KRR importance	0.55	0.89	0.06	0.06	0.01	0.96	0.99	0.03	0.08	0.00
		Linear MEM	0.13	0.90	0.05	0.01	0.00	0.29	0.99	0.04	0.06	0.00
		HIMA	0.13	0.84	0.05	0.02	0.00	0.28	1.00	0.01	0.02	0.00
		HDMA	0.14	0.85	0.06	0.02	0.00	0.27	1.00	0.03	0.03	0.00
		RegSEM	0.12	0.84	0.04	0.03	0.01	0.31	1.00	0.04	0.04	0.00

**Table 4:** Selection probabilities of candidate mediators under Mediator–Mediator Interaction Setting **S4**. Selection probabilities of candidate mediators across six methods (Kernel MEM, KRR importance, Linear MEM, HIMA, HDMA, and RegSEM) under scenario S4 with an interaction term between $M_{1}$ and $M_{4}$, for varying correlation among mediators (ρ), percentage of outcome variance unexplained by the exposure and true mediators ($Y_{\text{residual}}\%$), and sample size (*n*). $M_{1}$ and $M_{2}$ are true mediators; $M_{3}$ is an exposure-only semi-mediator; $M_{4}$ is an outcome-only semi-mediator; and “Others” refers to the null mediators $M_{5}-M_{22}$, which have no active causal pathway.

ρ	$Y_{\text{residual}}\%$	Method	*n* = 100					*n* = 200				
			$M_{1}$	$M_{2}$	$M_{3}$	$M_{4}$	Others	$M_{1}$	$M_{2}$	$M_{3}$	$M_{4}$	Others
0.0	75%	Kernel MEM	0.38	0.83	0.20	0.07	0.01	0.51	0.95	0.35	0.03	0.01
		KRR importance	0.12	0.56	0.08	0.07	0.01	0.31	0.87	0.09	0.05	0.00
		Linear MEM	0.06	0.86	0.06	0.03	0.00	0.03	0.97	0.04	0.02	0.00
		HIMA	0.02	0.68	0.01	0.02	0.00	0.02	0.95	0.03	0.00	0.00
		HDMA	0.05	0.87	0.06	0.04	0.00	0.07	0.97	0.06	0.02	0.00
		RegSEM	0.05	0.58	0.04	0.00	0.00	0.07	0.93	0.08	0.00	0.00
	50%	Kernel MEM	0.47	0.97	0.25	0.07	0.01	0.69	0.98	0.30	0.04	0.01
		KRR importance	0.35	0.88	0.09	0.13	0.01	0.90	0.99	0.04	0.21	0.00
		Linear MEM	0.06	0.95	0.04	0.05	0.00	0.08	0.97	0.06	0.02	0.00
		HIMA	0.04	0.92	0.01	0.04	0.00	0.04	0.97	0.03	0.01	0.00
		HDMA	0.11	0.94	0.09	0.04	0.00	0.11	0.97	0.09	0.02	0.00
		RegSEM	0.09	0.70	0.02	0.04	0.00	0.13	0.93	0.12	0.02	0.00
0.5	75%	Kernel MEM	0.31	0.76	0.25	0.07	0.01	0.51	0.99	0.39	0.03	0.00
		KRR importance	0.14	0.55	0.06	0.09	0.01	0.35	0.89	0.05	0.14	0.01
		Linear MEM	0.06	0.82	0.09	0.00	0.00	0.05	0.98	0.01	0.01	0.00
		HIMA	0.04	0.63	0.04	0.00	0.00	0.02	0.96	0.01	0.02	0.00
		HDMA	0.05	0.68	0.05	0.02	0.00	0.12	0.97	0.04	0.02	0.00
		RegSEM	0.05	0.65	0.05	0.01	0.00	0.05	0.98	0.07	0.00	0.00
	50%	Kernel MEM	0.46	0.91	0.18	0.07	0.01	0.75	1.00	0.30	0.06	0.00
		KRR importance	0.44	0.86	0.08	0.14	0.00	0.89	0.99	0.05	0.39	0.01
		Linear MEM	0.04	0.90	0.06	0.01	0.00	0.05	0.99	0.03	0.07	0.00
		HIMA	0.04	0.84	0.05	0.00	0.00	0.05	1.00	0.01	0.03	0.00
		HDMA	0.06	0.85	0.07	0.02	0.00	0.10	1.00	0.03	0.03	0.00
		RegSEM	0.07	0.87	0.08	0.02	0.00	0.11	1.00	0.09	0.02	0.00

**Table 5:** Summary of Mediator Selection. Superscripts identify other numbered or lettered rows reporting the same locus. HIMA and HDMA selections were based on the same screened datasets with 213 CpG loci.

Method	Loci Selection
1. Houtepen et al. (2016)	cg27512205, cg05608730^D^, cg26179948
2. van Kesteren and Oberski (2019)	cg02309301, cg12500973, cg16657538^A^, cg25626453^4,B^, cg13136721
3. Guo et al. (2022)	cg19230917^A^, cg06422529, cg03199124
4. Schaid and Sinnwell (2020)	cg01644731^A^, cg06890779, cg25626453^2,B^, cg26801646
A. Linear MEM-based Selection	cg16657538^2^, cg19230917^3^, cg01644731^4^, cg25448067, cg03025830, cg03643137, cg07343612^B^, cg16503628
B. Kernel MEM-based Selection	cg25626453^2,4^, cg07343612^A^, cg27103436
C. HIMA	cg10822172^D^
D. HDMA	cg05608730^1^, cg10822172^C^

**Fig. C1:**
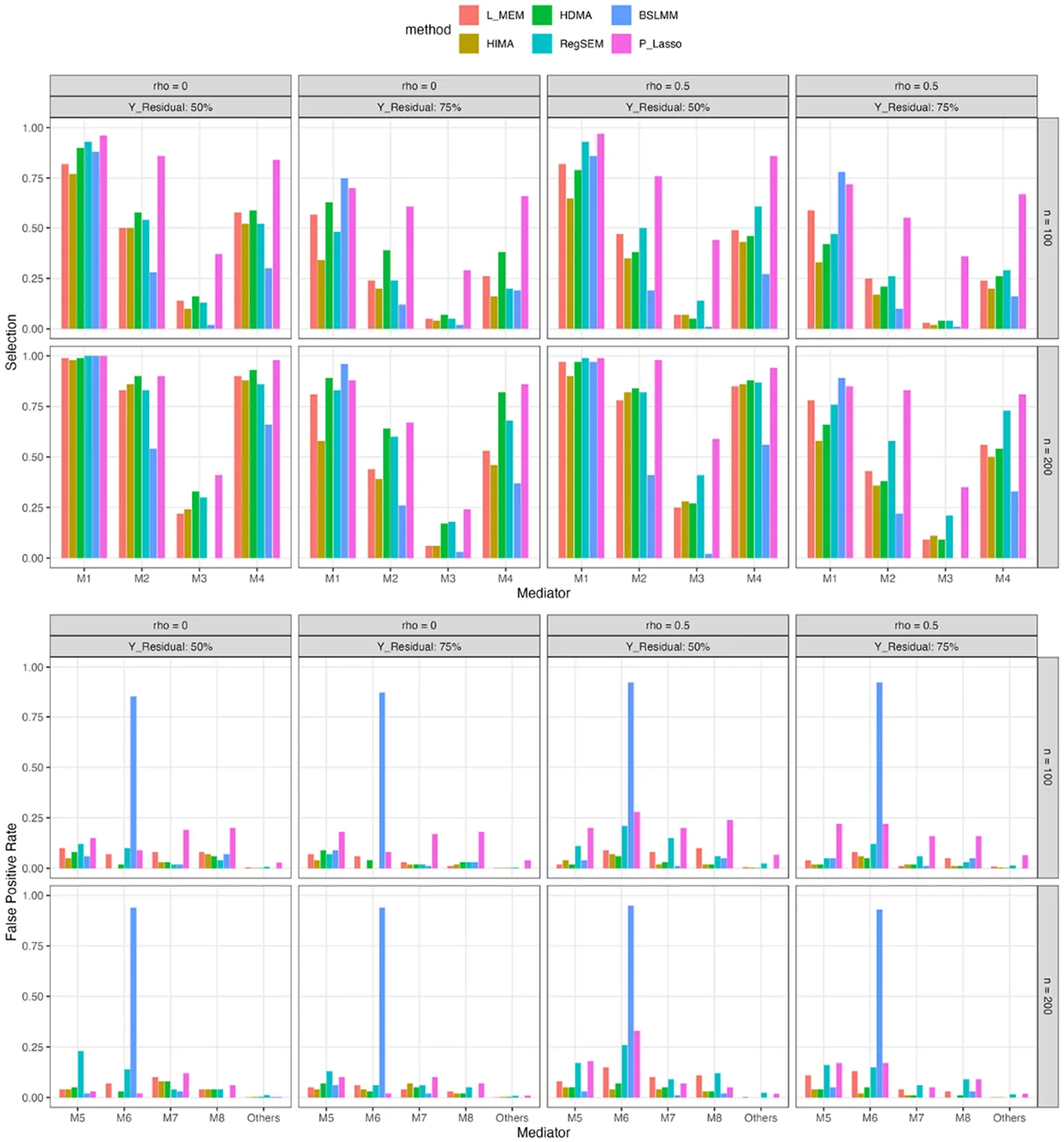
Full comparison under linear setting **S1**, including Pathway LASSO and BSLMM. Top panels report selection probabilities for the true mediators $M_{1}-M_{4}$; bottom panels report false positive rates for the semi-mediators $M_{5}-M_{8}$ and the null mediators (Others), over 100 Monte Carlo replications.

## Appendix A Algorithmic Details for the Kernel MEM-based Selection

### A.1 Characterization of the target set

**Proposition 1** *Suppose* Var(X)=1 *and that the linear model* (1a)-(1b) *holds, i.e.,* $M=\alpha X+\epsilon_{m}$ *and* $Y=\beta_{x}X+\beta_{m}^{T}M+\epsilon_{y}$. *Assume that* $\Sigma_{\mathrm{res}M}$ *is positive definite, that* $\epsilon_{m}∼𝒩(0,\Sigma_{\mathrm{res}M})$ *is independent of* X, *and* $E(\epsilon_{y}|X,M)=0$. *Let* h, $h_{-j}$, *and* $𝒮={𝒮}_{XM}\cap {𝒮}_{MY}$ *be defined as in* Section 2.1. *Then*

$$𝒮=\{j\;:\alpha_{j}\neq 0\;and\;\beta_{mj}\neq 0\}\;.$$

*Proof* As $E(M_{j}|X)=\alpha_{j}X$ and the variables are centered,

$$E[{\left\{E(M_{j}|X)-E(M_{j})\right\}}^{2}]=\alpha_{j}^{2}.$$

Thus $j\in {𝒮}_{XM}$ if and only if $\alpha_{j}\neq 0$. Moreover,

$$h(X,M)-h_{-j}(X,M_{-j})=\beta_{mj}\;\{M_{j}-E(M_{j}|X,M_{-j})\}\;.$$

Therefore,

$$E[{\left\{h(X,M)-h_{-j}(X,M_{j})\right\}}^{2}]=\beta_{mj}^{2}\text{Var}(M_{j}|X,M_{-j}).$$

Under Gaussian mediator errors, $\mathrm{Var}(M_{j}|X,M_{-j})$ does not depend on (X, $M_{-j}$), and positive definiteness of $\Sigma_{\mathrm{res}M}$ implies $\mathrm{Var}(M_{j}|X,M_{-j})={\left\{{\left(\Sigma_{\mathrm{res}M}^{-1}\right)}_{jj}\right\}}^{-1}>0$. The Gaussian assumption is needed only for this closed form. More generally, $E[{\left\{h(X,M)-h_{-j}(X,M_{-j})\right\}}^{2}]=\beta_{mj}^{2}E\{\mathrm{Var}(M_{j}|X,M_{-j})\}$, so the same conclusion holds whenever the expected conditional variance is positive. Hence $j\in {𝒮}_{MY}$ if and only if $\beta_{mj}\neq 0$, proving the result.

### A.2 Implementation Details for Kernel MEM-based selection

Algorithm 1 summarizes the proposed kernel MEM procedure. The exposure bandwidth is first estimated without penalization and then held fixed throughout mediator selection. For the X→M pathway, linear MEM is fitted with τ = 1, and the resulting allocation is converted to a selected set through nested top-*k* comparisons based on BIC. For the M→Y pathway, an initial unweighted pass is used to select the KRR penalty, followed by an adaptively weighted pass in which candidate mediator sets are compared using GCV. The sequential pruning rule with a tolerance of 10^−6^ is applied only to the outcome-pathway MEKRO fits.

Outcome-pathway selection requires a criterion that can compare candidate sets obtained under different budgets. For an unweighted Nadaraya–Watson fit with smoothing matrix $S_{\tau }$, we use the small-sample corrected Akaike information criterion (AICc) employed for MEKRO by White et al. (2017), ${\mathrm{AICc}}_{\tau }=\log({\hat{\mathrm{MSE}}}_{\tau })+(n+\nu_{\tau })∕(n-\nu_{\tau }-2)$, where ${\hat{\mathrm{MSE}}}_{\tau }$ is the in-sample mean squared error of the sparsified fit at budget τ and $\nu_{\tau }=\mathrm{tr}(S_{\tau })$ is the effective degrees of freedom. Fits with $\nu_{\tau }\geq n-2$ are treated as infeasible rather than scored. The first pass is unweighted and uses AICc only to obtain an initial outcome model for tuning $\alpha_{\mathrm{KRR}}$; the second pass applies the adaptive weights and compares candidate sets by GCV with $\alpha_{\mathrm{KRR}}$ held fixed. Thus AICc compares only fits that use the same unweighted specification.

**Table:**

Algorithm 1 Nonparametric Kernel MEM-based Mediator Selection
$$\begin{array}{cc} \; & \;\mathrm{Require}:\text{Standardized exposure}\;X,\;\text{mediators}\;M=\{M_{1},\ldots ,M_{p}\},\;\text{standardized}\\ \; & \;\;\;\text{outcome}\;Y;\;\text{coarse budget grid}\;{𝒯}_{c};\;\text{constants}\;\epsilon ,\gamma >0.\\ \; & \;\mathrm{Ensure}:\text{Selected mediator set}\;{ℳ}_{\text{selected}}.\\ \; & \;\;\;\mathrm{Step}\;0a:\mathrm{Fix}\;\mathrm{the}\;\mathrm{exposure}\;\mathrm{bandwidth}\\ \; & \;1:\text{Fit an unconstrained product-kernel Nadaraya}-\text{Watson regression by leave-one-out}\\ \; & \;\;\;\text{squared error and hold the resulting exposure bandwidth fixed to define}\;K_{X}.\\ \; & \;\;\;\mathrm{Step}\;0b:\mathrm{Compute}\;\mathrm{adaptive}\;\mathrm{weights}\\ \; & \;2:\text{Fit the mediator regressions and full and reduced KRR models};\;\text{compute and}\\ \; & \;\;\;\text{normalize}\;w_{j}\;\text{as in Section}\;3.2.\\ \; & \;\;\;\mathrm{Step}\;1:\mathrm{Select}\;\mathrm{active}\;X\to M\;\mathrm{pathways}\\ \; & \;3:\text{Optimize}\;\ell_{\hat{\text{SEL}}\;M|X}(\lambda )\;\text{using the reparameterization in Section}\;2.3\;\text{at}\;\tau =1.\\ \; & \;4:\text{Rank}\;\text{by}\;{\left\{{\hat{\lambda }}_{j}∕(1+{\hat{\lambda }}_{j})\right\}}^{1∕2};\;\text{let}\;{𝒮}_{XM}\;\text{be the nested top-}k\;\text{set with minimum}\\ \; & \;\;\;\text{mediatro-model BIC}.\\ \; & \;\;\;\mathrm{Step}\;2:\mathrm{Obtain}\;\mathrm{an}\;\mathrm{initial}\;\mathrm{outcome}\;\mathrm{model}\;\mathrm{and}\;\mathrm{tune}\;\mathrm{KRR}\\ \; & \;5:\text{For each}\;\tau \;\in \;{𝒯}_{c},\;\text{optimize unweighted MEKRO with}\;K_{X}\;\text{fixed},\;\text{sparsify},\;\text{and}\\ \; & \;\;\;{\text{compute AICc}}_{\tau }.\\ \; & \;6:{\text{Retain the set minimizing AICc}}_{\tau }\;\text{and tune}\;\alpha_{\text{KRR}}\;\text{by GCV on KRR refited to}\;X\\ \; & \;\;\;\text{and that mediator set}.\\ \; & \;\;\;\mathrm{Step}\;2b:\mathrm{Adaptively}\;\mathrm{weighted}\;\mathrm{outcome}\;\mathrm{selection}\\ \; & \;7:\text{For each}\;\tau \in {𝒯}_{c},\;\text{optimize weighted MEKRO with}\;K_{X}\;\text{fixed},\;\text{sparsify},\;\text{and compute}\\ \; & \;\;\;{\text{KRR GCV}}_{\tau }.\\ \; & \;8:\text{Form a fine grid around the best coarse}\;\tau ,\;\text{repeat the weighted fits},\;\text{and let}\;{𝒮}_{MY}\\ \; & \;\;\;\text{be the set with minimum GCV}.\\ \; & \;\;\;\mathrm{Step}\;3:\mathrm{Joint}\;\mathrm{selection}\\ \; & \;9:{ℳ}_{\text{selected}}\leftarrow {𝒮}_{XM}\cap {𝒮}_{MY}.\\ \; & \;10:\mathrm{return}\;{ℳ}_{\text{selected}} \end{array}$$

#### Computational settings.

For the initial unpenalized outcome regression with d standardized predictors, all bandwidths are initialized at $n^{-1∕(d+4)}$. Bandwidths are optimized on the log scale using L-BFGS-B over [0.05, 20], with a convergence tolerance of 10^−6^ and a maximum of 200 iterations. No random restarts are used. Any finite solution is retained regardless of the optimizer’s reported convergence status. If either the returned parameter vector or the objective value is nonfinite, the initial bandwidth vector is used.

Outcome-pathway selection is performed over a coarse grid of 30 equally spaced budget values from 0.01 to 12. A fine grid of 10 equally spaced values is then constructed over one coarse-grid interval on either side of the optimal coarse-grid value, subject to a lower bound of 0.01. The KRR penalty is selected from 20 logarithmically spaced values between 10^−12^ and 10^2^. Each MEKRO fit is initialized at $v=1_{p}$ and optimized using L-BFGS-B with a convergence tolerance of 10^−7^ and no random restarts. The returned parameter vector is retained regardless of the optimizer’s reported convergence status.

## Appendix B Simulation Details for Linear Setting with Random Coefficients

We extended the linear simulation by drawing the pathway coefficients at random and increasing the mediator dimension to *p* = 50. Data generation otherwise followed Section 4.1: X∼N(0,1), $M=\alpha X+\epsilon_{m}$, and $\epsilon_{m}$ was multivariate Gaussian with common residual variance $\sigma_{M}^{2}$ and either independent components or a permuted AR(1) correlation structure with ρ = 0.5. We considered n∈{100,200} and conducted 100 Monte Carlo replications for each configuration. Mediators $M_{1}-M_{4}$ were active in both pathways; $M_{5}$ and $M_{6}$ were active only in the X→M pathway; $M_{7}$ and $M_{8}$ were active only in the M→Y pathway; and $M_{9}-M_{50}$ were inactive in both pathways.

The active coefficients $\alpha_{j}$ and $\beta_{mj}$ were drawn independently from signed two-component mixture distributions, with signs assigned independently and with equal probability. Consequently, the two pathway coefficients of a true mediator had probability 0.5 of being drawn from different magnitude components, generating pathway imbalance across replications. In both signal scenarios, $|\beta_{mj}|$ was drawn with equal probability from 𝒰(0.3,0.6) or 𝒰(0.8,1.2). In the low-signal scenario, $\left(\sigma_{M}^{2},\sigma_{Y}^{2})=(3,54\right)$ and $|\alpha_{j}|$ was drawn with equal probability from 𝒰(0.15,0.40) or 𝒰(0.65,1.05), yielding $R_{M}^{2}\approx 0.11$ and $R_{Y}^{2}\approx 0.19$. In the high-signal scenario, $\left(\sigma_{M}^{2},\sigma_{Y}^{2})=(1,14\right)$ and $|\alpha_{j}|$ was drawn from 𝒰(0.15,0.40) or 𝒰(0.65,0.95), yielding $R_{M}^{2}\approx 0.23$ and $R_{Y}^{2}\approx 0.27$. Here, $R_{M}^{2}$ and $R_{Y}^{2}$ denote the proportions of mediator and outcome variance, respectively, explained by models (1a) and (1b). After standardization, the expected coefficient magnitudes in the low-signal scenario were ${\tilde{\alpha }}_{j}\approx 0.30$, ${\tilde{\beta }}_{mj}\approx 0.16$, and ${\tilde{\beta }}_{x}\approx 0.12$; the corresponding values in the high-signal scenario were ${\tilde{\alpha }}_{j}\approx 0.44$, ${\tilde{\beta }}_{mj}\approx 0.19$, and ${\tilde{\beta }}_{x}\approx 0.22$. A tilde denotes a coefficient from models (1a)-(1b) after standardization.

We compared linear MEM with HIMA, HDMA, and RegSEM. Because the coefficients were redrawn in each replication, selection probabilities were averaged within the four structural groups rather than reported for individual mediators: true mediators ($M_{1}-M_{4}$), exposure-only semi-mediators ($M_{5}$ and $M_{6}$), outcome-only semi-mediators ($M_{7}$ and $M_{8}$), and null mediators ($M_{9}-M_{50}$). Table B1 reports the results.

**Table B1:** Simulation results for the linear setting with random coefficients (*p* = 50). Entries are selection probabilities averaged within each structural group over 100 Monte Carlo replications: true mediators (True), exposure-only semi-mediators (X–M), outcome-only semi-mediators (M–Y), and null mediators (Null).

ρ	($\alpha_{M}^{2}$, $\alpha_{Y}^{2}$)	Method	*n* = 100				*n* = 200			
			True	X–M	M–Y	Null	True	X–M	M–Y	Null
0.0	(1, 14)	Linear MEM	0.39	0.08	0.03	0.01	0.60	0.04	0.03	0.02
		HIMA	0.22	0.04	0.03	0.02	0.47	0.01	0.03	0.03
		HDMA	0.40	0.08	0.03	0.03	0.62	0.08	0.03	0.05
		RegSEM	0.21	0.03	0.01	0.01	0.50	0.03	0.00	0.01
0.0	(3, 54)	Linear MEM	0.27	0.07	0.03	0.01	0.45	0.03	0.02	0.01
		HIMA	0.15	0.03	0.03	0.01	0.34	0.00	0.03	0.02
		HDMA	0.28	0.06	0.02	0.03	0.50	0.04	0.03	0.04
		RegSEM	0.14	0.03	0.03	0.01	0.31	0.01	0.01	0.01
0.5	(1, 14)	Linear MEM	0.37	0.07	0.02	0.01	0.54	0.05	0.04	0.02
		HIMA	0.25	0.04	0.01	0.03	0.41	0.02	0.01	0.03
		HDMA	0.33	0.03	0.01	0.05	0.53	0.04	0.01	0.04
		RegSEM	0.19	0.00	0.01	0.01	0.43	0.03	0.01	0.01
0.5	(3, 54)	Linear MEM	0.27	0.05	0.02	0.01	0.40	0.06	0.03	0.01
		HIMA	0.18	0.04	0.01	0.02	0.29	0.03	0.00	0.03
		HDMA	0.22	0.03	0.00	0.05	0.39	0.04	0.01	0.03
		RegSEM	0.12	0.00	0.01	0.00	0.28	0.03	0.01	0.01

Selection of true mediators followed the pattern observed in setting **S1**. With independent mediator residuals (ρ = 0), HDMA and linear MEM had the highest selection probabilities and performed similarly. Under the high-signal configuration, for example, the probabilities were 0.40 and 0.39, respectively, at *n* = 100, and 0.62 and 0.60 at *n* = 200. With correlated residuals (ρ = 0.5), linear MEM had slightly higher true-mediator selection probabilities than HDMA in most configurations, including 0.37 versus 0.33 at *n* = 100 under high signal. This reversal is consistent with the joint modeling of correlated mediators in the MEM criterion. HIMA and RegSEM generally had lower true-mediator selection probabilities, although RegSEM improved appreciably at *n* = 200.

For every method, true-mediator selection increased with signal strength and sample size. Selection rates were uniformly higher under ($\sigma_{M}^{2}$, $\sigma_{Y}^{2}$) = (1, 14) than under (3, 54), and the increase from *n* = 100 to *n* = 200 was most pronounced in the low-signal scenario. Exposure-only and outcome-only semi-mediators were selected at rates no greater than 0.08, and null-mediator selection did not exceed 0.05. HDMA reached the upper end of the null-selection range in several configurations, whereas linear MEM and RegSEM generally had lower null-selection rates.

Thus, when pathway coefficients were random and potentially imbalanced, linear MEM performed similarly to HDMA under independent mediator residuals and had a modest advantage under correlated residuals. The remaining semi-mediator selections indicate that pathway imbalance continues to complicate separation of variables active in only one pathway from true mediators.

## Appendix C Additional Comparisons for the Linear Setting S1

Figure C1 presents additional comparisons with Pathway LASSO (Zhao and Luo 2022) and BSLMM (Song et al. 2020). For Pathway LASSO, hyperparameters were tuned only for one dataset and used for all simulations in the same setting because the tuning procedure was computationally intensive. Across all settings, Pathway LASSO exhibited consistently inflated false positive rates, both overall and among semi-mediators, a pattern also observed by Clark-Boucher et al. (2023), and attributed to its relatively stronger penalization of true mediators than semi-mediators (Jones et al. 2024).

In contrast, BSLMM was highly sensitive to the choice of posterior inclusion probability (PIP) thresholds. Across different simulation settings, the median PIP was 0.0018 for true mediators, with an interquartile range (IQR) of 0.0006 to 0.0066, whereas semi-mediators and non-mediators had substantially lower PIP values, with a median of 0.0002 and an IQR of 0.0000 to 0.0004. Although some separation between true mediators and semi-/non-mediators was evident, the PIP values were far below commonly adopted thresholds such as 0.10, rendering threshold selection nontrivial. In the accompanying figure, we therefore calibrated the PIP threshold to control the expected number of false positives to one per simulation rather than applying a fixed cutoff. Under this calibration, the resulting false positive rate remained substantially higher than that of other methods (linear MEM, HIMA, HDMA, and RegSEM), limiting the interpretability of direct performance comparisons.^1^

1 The excess was concentrated almost entirely on the exposure-only semi-mediator $M_{8}$, whose selection rate under BSLMM reached 0.82 to 0.98 across configurations, while its rate among the null mediators remained near zero.

## Appendix D Robustness to Distributional Misspecification

The MEM selection likelihood is built on a Gaussian working model, so it is natural to ask how much of its behavior depends on that assumption holding. We therefore assessed the proposed linear MEM-based approach under violations of the Gaussian residual assumption. We considered a simplified version of the linear setting with homogeneous mediation effects, where p = 22, $\alpha ={\left(1,1,1,1,0.5,0.5,0,0,0,\ldots ,0\right)}^{\prime }$ and $\beta_{m}={\left(1,1,1,1,0,0,0.5,0.5,0,\ldots ,0\right)}^{\prime }$, so that $M_{1}-M_{4}$ were true mediators, $M_{5}$ and $M_{6}$ exposure-only semi-mediators, $M_{7}$ and $M_{8}$ outcome-only semi-mediators, and $M_{9}-M_{22}$ null, as in **S1**. For the active mediators the noise was scaled by the same coefficient as the exposure signal, so the exposure-only semi-mediators carried the same X→M signal-to-noise ratio as the true mediators; this makes them the hardest group to screen out and is worth bearing in mind when reading the ${\mathrm{FP}}_{XM}$ column.

Non-Gaussian variates were used for the exposure as well as for the mediator and outcome residuals, so that the whole system, and not only the error terms, departed from Gaussianity. Three residual noise distributions were examined: (1) a skewed-normal distribution with shape parameter a = 2; (2) a skewed-normal distribution with shape parameter a = 4; and (3) a Gaussian mixture distribution, with 15% of residuals drawn from a component with mean 2 and 85% from a component with mean 0. In each case the draws were centered and scaled to empirical mean zero and unit variance before entering the models, so that the distributional shape, and not the noise level, was the quantity being varied.

Throughout this appendix the mediator residual was held at a level corresponding to a 90% mediator-residual share, while the outcome residual share was set to either 90% or 50%; mediator residuals were given either an independent or a permuted AR(1) structure, with the permutation redrawn in every replication, and the mediators were standardized only after the full data-generating process. One further difference from **S1** should be noted: the exposure coefficient here was set so that the direct effect contributed variance equal to that of the mediator component, rather than being calibrated to 10% of the systematic variation. Together with the different coefficient values, this means the results below are not on the same footing as Table 1 and should not be read against it. To obtain a distribution-matched reference, we repeated this design using standard Gaussian draws for the exposure, the independent mediator innovations before imposing the residual-correlation structure, and the outcome error. All coefficient values, variance settings, correlation structures, permutation and standardization procedures, and sample sizes were unchanged, and the same set of Monte Carlo seeds was used.

We summarized the selection probability of true mediators (TP), the overall false positive rate (FP), and the false positive rate specific to semi-mediators in Tables D1-D4. Here TP is the average selection rate over the true mediators $M_{1}-M_{4}$, ${\mathrm{FP}}_{MY}$ that over the outcome-only semi-mediators $M_{5}$ and $M_{6}$, ${\mathrm{FP}}_{XM}$ that over the exposure-only semi-mediators $M_{7}$ and $M_{8}$, and FP that over the null mediators $M_{9}-M_{22}$.

The Gaussian reference showed that the relative performance of the methods already depended on the signal strength and mediator residual correlation when the working distribution was correctly specified. HDMA generally had the highest true-mediator selection probability under independent mediator residuals, whereas its advantage diminished under correlated residuals. RegSEM frequently had the highest true-mediator selection probability when the outcome signal was stronger and *n* = 200, but it also selected the exposure-only semi-mediators more often than the other methods. These patterns persisted across the three non-Gaussian distributions and therefore cannot be attributed solely to distributional misspecification.

For linear MEM, the true-mediator selection probability across the 24 matched non-Gaussian comparisons averaged 0.01 below the Gaussian reference. The cell-specific difference ranged from a decrease of 0.07 to an increase of 0.02. ${\mathrm{FP}}_{MY}$ differed by at most 0.01, and ${\mathrm{FP}}_{XM}$ ranged from 0.05 lower to 0.05 higher than its Gaussian counterpart, with no systematic increase on average. The null-mediator false positive rate increased by less than 0.01 on average and by at most 0.03. Thus, the examined departures from Gaussianity produced a modest reduction in true-mediator selection for linear MEM rather than a large or uniform deterioration, while false selections remained uncommon. This conclusion is limited to the skewed-normal and Gaussian-mixture distributions and signal configurations considered here.

**Table D1:** Matched Gaussian Reference for the Distributional Robustness Analysis

ρ	$Y_{\text{residual}}\%$	Method	*n* = 100				*n* = 200			
			TP	${\mathrm{FP}}_{MY}$	${\mathrm{FP}}_{XM}$	FP	TP	${\mathrm{FP}}_{MY}$	${\mathrm{FP}}_{XM}$	FP
0.0	90%	Linear MEM	0.20	0.01	0.09	0.00	0.24	0.00	0.07	0.00
		HIMA	0.08	0.01	0.01	0.00	0.14	0.00	0.01	0.00
		HDMA	0.19	0.01	0.04	0.00	0.37	0.00	0.08	0.01
		RegSEM	0.07	0.00	0.01	0.00	0.27	0.00	0.09	0.00
0.0	50%	Linear MEM	0.46	0.00	0.02	0.00	0.86	0.00	0.03	0.00
		HIMA	0.39	0.01	0.03	0.00	0.88	0.01	0.04	0.00
		HDMA	0.57	0.01	0.04	0.00	0.96	0.00	0.08	0.00
		RegSEM	0.33	0.01	0.05	0.01	0.91	0.01	0.20	0.01
0.5	90%	Linear MEM	0.17	0.01	0.12	0.00	0.24	0.00	0.10	0.00
		HIMA	0.08	0.01	0.01	0.00	0.13	0.01	0.04	0.00
		HDMA	0.11	0.00	0.04	0.00	0.18	0.01	0.09	0.00
		RegSEM	0.08	0.00	0.01	0.00	0.25	0.00	0.07	0.01
0.5	50%	Linear MEM	0.42	0.01	0.02	0.00	0.81	0.01	0.05	0.00
		HIMA	0.35	0.01	0.03	0.00	0.74	0.01	0.04	0.00
		HDMA	0.42	0.00	0.04	0.00	0.80	0.01	0.08	0.00
		RegSEM	0.42	0.01	0.07	0.01	0.90	0.01	0.20	0.02

**Table D2:** Skewed-normal Residual Error with α = 2

ρ	$Y_{\text{residual}}\%$	Method	*n* = 100				*n* = 200			
			TP	${\mathrm{FP}}_{MY}$	${\mathrm{FP}}_{XM}$	FP	TP	${\mathrm{FP}}_{MY}$	${\mathrm{FP}}_{XM}$	FP
0.0	90%	Linear MEM	0.17	0.01	0.08	0.02	0.21	0.00	0.08	0.01
		HIMA	0.06	0.00	0.01	0.00	0.10	0.00	0.00	0.00
		HDMA	0.19	0.00	0.04	0.01	0.31	0.00	0.04	0.01
		RegSEM	0.06	0.00	0.01	0.00	0.19	0.01	0.04	0.01
0.0	50%	Linear MEM	0.47	0.00	0.02	0.00	0.84	0.00	0.01	0.00
		HIMA	0.42	0.00	0.02	0.00	0.86	0.00	0.02	0.00
		HDMA	0.62	0.00	0.04	0.01	0.93	0.01	0.04	0.01
		RegSEM	0.32	0.00	0.04	0.01	0.90	0.03	0.16	0.03
0.5	90%	Linear MEM	0.16	0.00	0.11	0.02	0.21	0.01	0.07	0.01
		HIMA	0.07	0.00	0.03	0.00	0.09	0.00	0.02	0.00
		HDMA	0.08	0.00	0.04	0.01	0.14	0.01	0.04	0.01
		RegSEM	0.06	0.00	0.04	0.01	0.18	0.00	0.05	0.02
0.5	50%	Linear MEM	0.44	0.00	0.03	0.01	0.74	0.00	0.03	0.01
		HIMA	0.34	0.00	0.04	0.01	0.71	0.02	0.03	0.01
		HDMA	0.41	0.00	0.04	0.01	0.75	0.01	0.03	0.00
		RegSEM	0.46	0.04	0.06	0.02	0.86	0.03	0.14	0.04

**Table D3:** Skewed-normal Residual Error with α = 4

ρ	$Y_{\text{residual}}\%$	Method	*n* = 100				*n* = 200			
			TP	${\mathrm{FP}}_{MY}$	${\mathrm{FP}}_{XM}$	FP	TP	${\mathrm{FP}}_{MY}$	${\mathrm{FP}}_{XM}$	FP
0.0	90%	Linear MEM	0.17	0.00	0.09	0.02	0.23	0.00	0.10	0.01
		HIMA	0.07	0.00	0.03	0.01	0.11	0.00	0.01	0.00
		HDMA	0.17	0.00	0.06	0.01	0.32	0.01	0.07	0.01
		RegSEM	0.07	0.01	0.02	0.01	0.25	0.00	0.07	0.01
0.0	50%	Linear MEM	0.47	0.00	0.05	0.01	0.87	0.00	0.02	0.00
		HIMA	0.39	0.00	0.01	0.00	0.87	0.01	0.06	0.01
		HDMA	0.61	0.00	0.06	0.01	0.95	0.01	0.06	0.01
		RegSEM	0.34	0.00	0.06	0.01	0.87	0.01	0.16	0.02
0.5	90%	Linear MEM	0.15	0.02	0.11	0.03	0.23	0.01	0.09	0.01
		HIMA	0.06	0.00	0.05	0.01	0.09	0.01	0.03	0.00
		HDMA	0.09	0.00	0.06	0.01	0.16	0.01	0.04	0.01
		RegSEM	0.10	0.00	0.04	0.01	0.20	0.00	0.07	0.02
0.5	50%	Linear MEM	0.39	0.01	0.04	0.01	0.76	0.01	0.04	0.01
		HIMA	0.32	0.00	0.04	0.01	0.73	0.00	0.04	0.01
		HDMA	0.40	0.00	0.06	0.01	0.78	0.01	0.03	0.01
		RegSEM	0.48	0.02	0.09	0.02	0.88	0.03	0.15	0.03

**Table D4:** Mixed Gaussian Distribution Error

ρ	$Y_{\text{residual}}\%$	Method	*n* = 100				*n* = 200			
			TP	${\mathrm{FP}}_{MY}$	${\mathrm{FP}}_{XM}$	FP	TP	${\mathrm{FP}}_{MY}$	${\mathrm{FP}}_{XM}$	FP
0.0	90%	Linear MEM	0.18	0.00	0.10	0.02	0.24	0.00	0.05	0.01
		HIMA	0.08	0.00	0.02	0.00	0.14	0.00	0.00	0.00
		HDMA	0.19	0.00	0.04	0.01	0.33	0.00	0.05	0.01
		RegSEM	0.06	0.00	0.01	0.00	0.29	0.00	0.04	0.01
0.0	50%	Linear MEM	0.45	0.00	0.01	0.00	0.84	0.00	0.01	0.00
		HIMA	0.42	0.00	0.01	0.00	0.87	0.00	0.02	0.00
		HDMA	0.62	0.00	0.04	0.01	0.94	0.01	0.03	0.01
		RegSEM	0.36	0.00	0.06	0.02	0.92	0.01	0.16	0.03
0.5	90%	Linear MEM	0.17	0.00	0.10	0.02	0.24	0.00	0.05	0.01
		HIMA	0.09	0.00	0.04	0.01	0.11	0.00	0.01	0.00
		HDMA	0.10	0.00	0.06	0.01	0.19	0.00	0.03	0.01
		RegSEM	0.06	0.00	0.04	0.01	0.25	0.01	0.03	0.01
0.5	50%	Linear MEM	0.43	0.01	0.07	0.01	0.79	0.00	0.01	0.00
		HIMA	0.34	0.00	0.04	0.01	0.69	0.00	0.02	0.00
		HDMA	0.43	0.00	0.05	0.01	0.77	0.00	0.03	0.00
		RegSEM	0.43	0.02	0.10	0.03	0.90	0.03	0.15	0.04

## List of Abbreviations

AICc: Corrected Akaike information criterion
AR(1): First-order autoregressive
BIC: Bayesian information criterion
BSLMM: Bayesian sparse linear mixed model
CpG: Cytosine-phosphate-guanine dinucleotide
DNA: Deoxyribonucleic acid
GCV: Generalized cross-validation
HDMA: High-dimensional mediation analysis (de-sparsified LASSO)
HIMA: High-dimensional mediation analysis (minimax concave penalty)
IQR: Interquartile range
KRR: Kernel ridge regression
LASSO: Least absolute shrinkage and selection operator
L-BFGS: Limited-memory Broyden-Fletcher-Goldfarb-Shanno algorithm (with box constraints)
MedFix: Mediation analysis with fixed effect model
MEKRO: Measurement error kernel regression operator
MEM: Measurement error modeling
MSE: Mean squared error
PIP: Posterior inclusion probability
RegSEM: Regularized structural equation model
TP, FP: True positive, false positive
